# Deferasirox, an oral iron chelator, with gemcitabine synergistically inhibits pancreatic cancer cell growth *in vitro* and *in vivo*

**DOI:** 10.18632/oncotarget.25421

**Published:** 2018-06-19

**Authors:** Shuhei Shinoda, Seiji Kaino, Shogo Amano, Hirofumi Harima, Toshihiko Matsumoto, Koichi Fujisawa, Taro Takami, Naoki Yamamoto, Takahiro Yamasaki, Isao Sakaida

**Affiliations:** ^1^ Department of Gastroenterology and Hepatology, Yamaguchi University Graduate School of Medicine, Yamaguchi, Japan; ^2^ Department of Oncology and Laboratory Medicine, Yamaguchi University, Graduate School of Medicine, Yamaguchi, Japan

**Keywords:** deferasirox, iron chelator, gemcitabine, pancreatic cancer, ribonucleotide reductase

## Abstract

**Objectives:**

Iron is an essential element for cell proliferation and growth processes. We have reported that deferasirox (DFX), an oral iron chelator, showed antiproliferative activity against pancreatic cancer cells. This study aimed to elucidate the effects of combination of gemcitabine (GEM), standard chemotherapy for pancreatic cancer, and DFX *in vitro* and *in vivo*.

**Results:**

GEM+DFX showed antiproliferative activity and induced apoptosis in pancreatic cancer cells *in vitro*. GEM+DFX suppressed xenograft tumor growth and induced apoptosis without any serious side effects compared with control, GEM, and DFX (average tumor volume: control 697 mm^3^ vs GEM 372 mm^3^, *p* < 0.05; GEM 372 mm^3^ vs GEM+DFX 234 mm^3^, *p* < 0.05). RRM1 and RRM2 protein levels were substantially reduced by DFX in BxPC-3 *in vitro*.

**Conclusion:**

GEM+DFX has significant anticancer effects on pancreatic cancer cell through RR activity suppression.

**Methods:**

BxPC-3, a human pancreatic cancer cell line, was used in all experiments. Cellular proliferation rate was measured using 3-(4,5-dimethylthiazol-2-yl)-5-(3-carboxymethoxyphenyl)-2-(4-sulfophenyl)-2H-tetrazolium, inner salt assay. Apoptosis was evaluated by flow cytometry and by measuring caspase 3/7 activity with luminescence assay. In the tumor xenografts in nude mice models, when five weeks after engraftment, drug administration began (day 0). After treatment for 21 days, the mice were sacrificed and the tumors were excised. Apoptotic cells in xenografts were evaluated by terminal deoxynucleotidyl transferase deoxyuridine triphosphate nick-end labeling assay. Protein levels of ribonucleotide reductase (RR) subunit 1 (RRM1) and RR subunit 2 (RRM2) in BxPC-3 cells were assessed by western blot *in vitro*.

## INTRODUCTION

Pancreatic cancer is a highly lethal disease, in which mortality closely parallels incidence. The 5-year survival rate of pancreatic cancer, including resectable cases, is not more than 10% [1]. For patients with unresectable pancreatic cancer, chemotherapy is the mainstay of treatment. During the last two decades, gemcitabine (GEM), a nucleoside analog of deoxycytidine, has been the standard chemotherapeutic agent for pancreatic cancer [2]. Recently, new combination chemotherapies, such as regimens combining fluorouracil, irinotecan, oxaliplatin, and leucovorin (FOLFIRINOX) [3] or albumin-bound paclitaxel with GEM [4], have been reported. However, while combination chemotherapies have shown therapeutic advantages over single-agent GEM, they also have a high incidence of side effects. Moreover, more than half of pancreatic cancer patients are diagnosed at an age of ≥65 years [5]. Hence, GEM remains a key drug, and a new chemotherapeutic strategy for pancreatic cancer is still required, especially for those with refractory cancer, because of side effects and/or advanced age.

Iron is an essential element for cell proliferation and growth processes [5]. Iron chelators, which are commonly used for iron-overload disease, have shown antiproliferative effects for numerous kinds of cancer [6]. Additionally, iron chelators have lower incidence of side effects because they are not classified as anticancer drugs. We have conducted the world’s first pilot study of deferoxamine (DFO) therapy in patients with advanced hepatocellular carcinoma and subsequently reported the efficacy of this approach [7]. However, DFO cannot be administered orally, thereby limiting its clinical application. Recently, deferasirox (DFX), a new oral iron chelator, has been developed. We found that DFX has antiproliferative activity against pancreatic cancer cells *in vitro* and *in vivo*; however, DFX did not induce apoptosis sufficiently [8]. We have also reported that the combination of DFX and sorafenib exerts a stronger inhibitory effect in hepatocarcinogenesis compared with DFX alone [9]. Against this background, we evaluated the combination of GEM and DFX against pancreatic cancer for the first time. Here, we showed the antiproliferative activity of the combined treatment of GEM+DFX against pancreatic cancer cells.

## RESULTS

### Antiproliferative activity of GEM+DFX against pancreatic cancer cells *in vitro*

**Figure 1:**
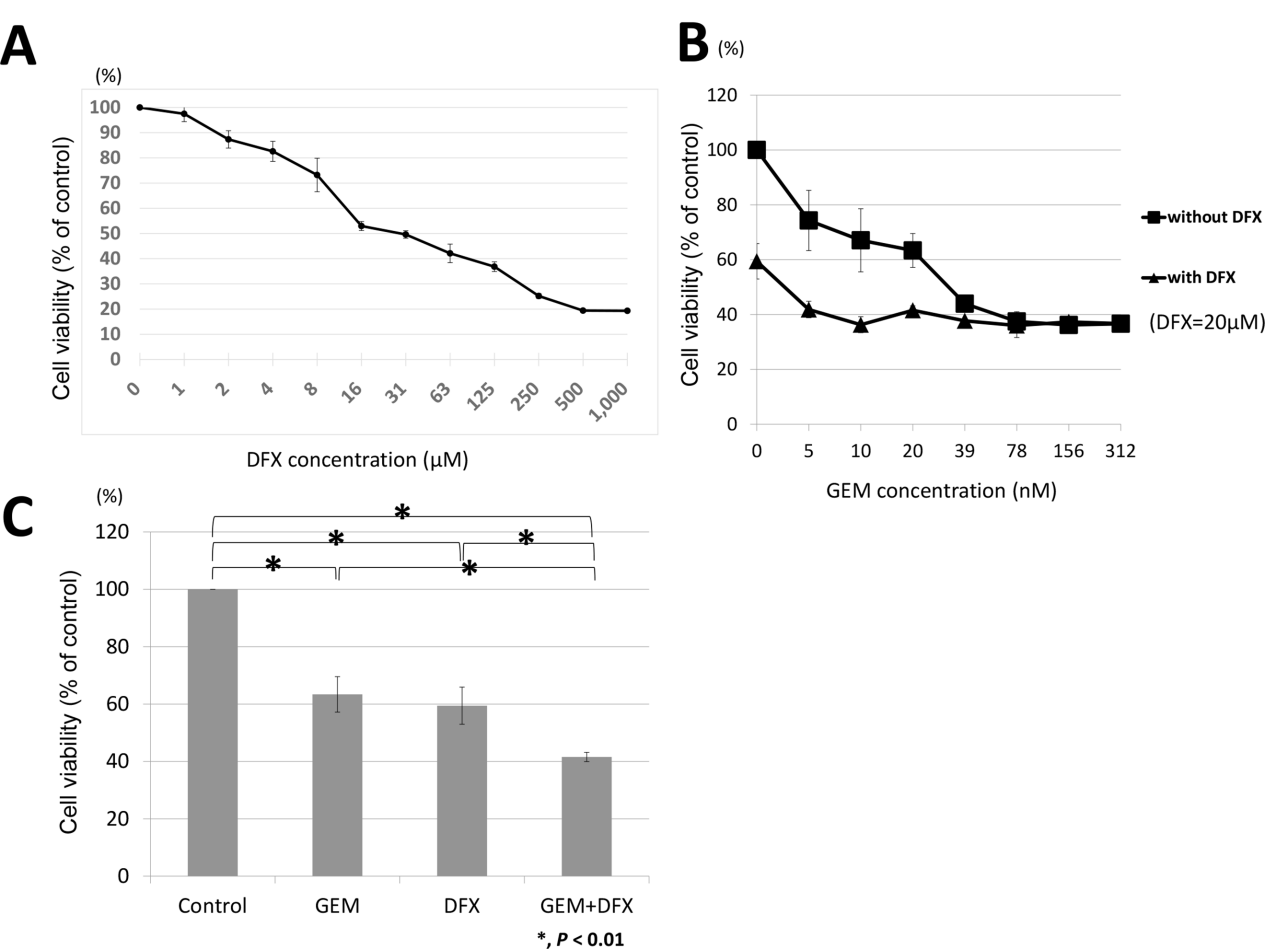
GEM+DFX has antiproliferative activity against pancreatic cancer cells *in vitro* (**A**) DFX inhibited the proliferation of BxPC-3 cells. The cells were treated with DFX for 72 h. The viability of BxPC-3 cells incubated with DFX decreased in a dose-dependent manner. Data are presented as mean ± SD (*n* = 3). (**B**) GEM and GEM+DFX inhibited the proliferation of BxPC-3 cells. The cells were treated with GEM and/or DFX for 72 h. The viability of BxPC-3 cells incubated with GEM and/or DFX decreased in a dose-dependent manner. Data are presented as mean ± SD (*n* = 3). (**C**) The antiproliferative activity of the combined treatment of 20 nM GEM and 20 μM DFX for BxPC-3 was significantly higher than that of GEM alone. Data are presented as mean ± SD (*n* = 3).

BxPC-3 was incubated with either the vehicle control or the indicated concentrations of DFX for 72 h; subsequently, cell survival rates were measured using the 3-(4,5-dimethylthiazol-2-yl)-5-(3-carboxymethoxyphenyl)-2-(4-sulfophenyl)-2H-tetrazolium, inner salt (MTS) assay. The survival rate of cells decreased in a dose-dependent manner when treated with DFX (Figure 1A). Then, we examined the antiproliferative activity of the combined treatment of GEM+DFX against BxPC-3. As shown in Figure 1, the IC50 value of DFX in BxPC-3 was 22.1 ± 2.1 μM. Thus, BxPC-3 was exposed to GEM (0, 5, 10, 20, 39, 78, 156, 312 nM) with 20 μM DFX or without DFX for 72 h. The cell survival rates are shown in Figure 1B. The antiproliferative activity of the combined treatment of 20 nM GEM and 20 μM DFX was significantly higher than that of GEM alone (Figure 1C).

### Apoptosis in pancreatic cancer cells treated with GEM + DFX *in vitro*

**Figure 2:**
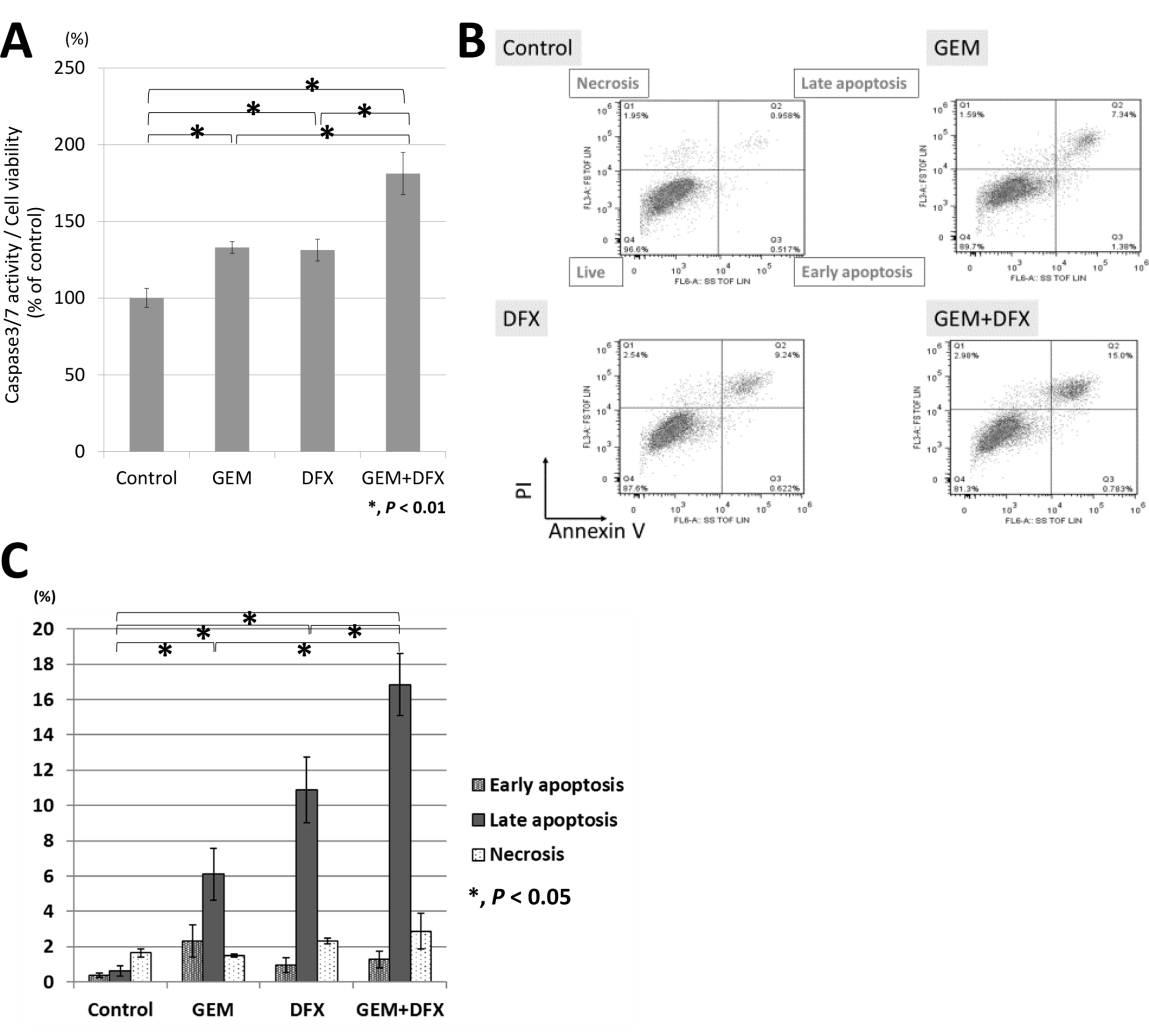
GEM+DFX induced apoptosis in pancreatic cancer cells *in vitro* (**A**) The caspase 3/7 activity of the combined treatment of GEM and DFX for BxPC-3 was significantly higher than that of Gem alone. Data are presented as mean ± SD (control group: *n* = 6, GEM group: *n* = 3, DFX group: *n* = 3, GEM+DFX group: *n* = 3). (**B**) GEM, DFX, and GEM+DFX induced apoptosis in BxPC-3. (**C**) The number of late apoptosis cells of the combined treatment of GEM and DFX for BxPC-3 was significantly higher than that of GEM alone. Data are presented as the mean ± SD (*n* = 3).

Apoptosis was evaluated by measuring caspase 3/7 activity with luminescence assay. Considering the results of cell proliferation assay, we added 20 nM GEM, 20 μM DFX, or 20 nM GEM and 20 μM DFX to each well. The caspase 3/7 activity of the combined treatment of GEM+DFX was significantly higher than that of GEM alone (Figure 2A). Flow cytometry using PI and Annexin V staining was employed in the apoptosis analysis. The number of late apoptosis cells of the combined treatment of GEM+DFX was significantly higher than that of GEM alone (Figure 2B, 2C).

### Tumor growth suppression and apoptosis without serious side effects *in vivo* by GEM + DFX

**Figure 3:**
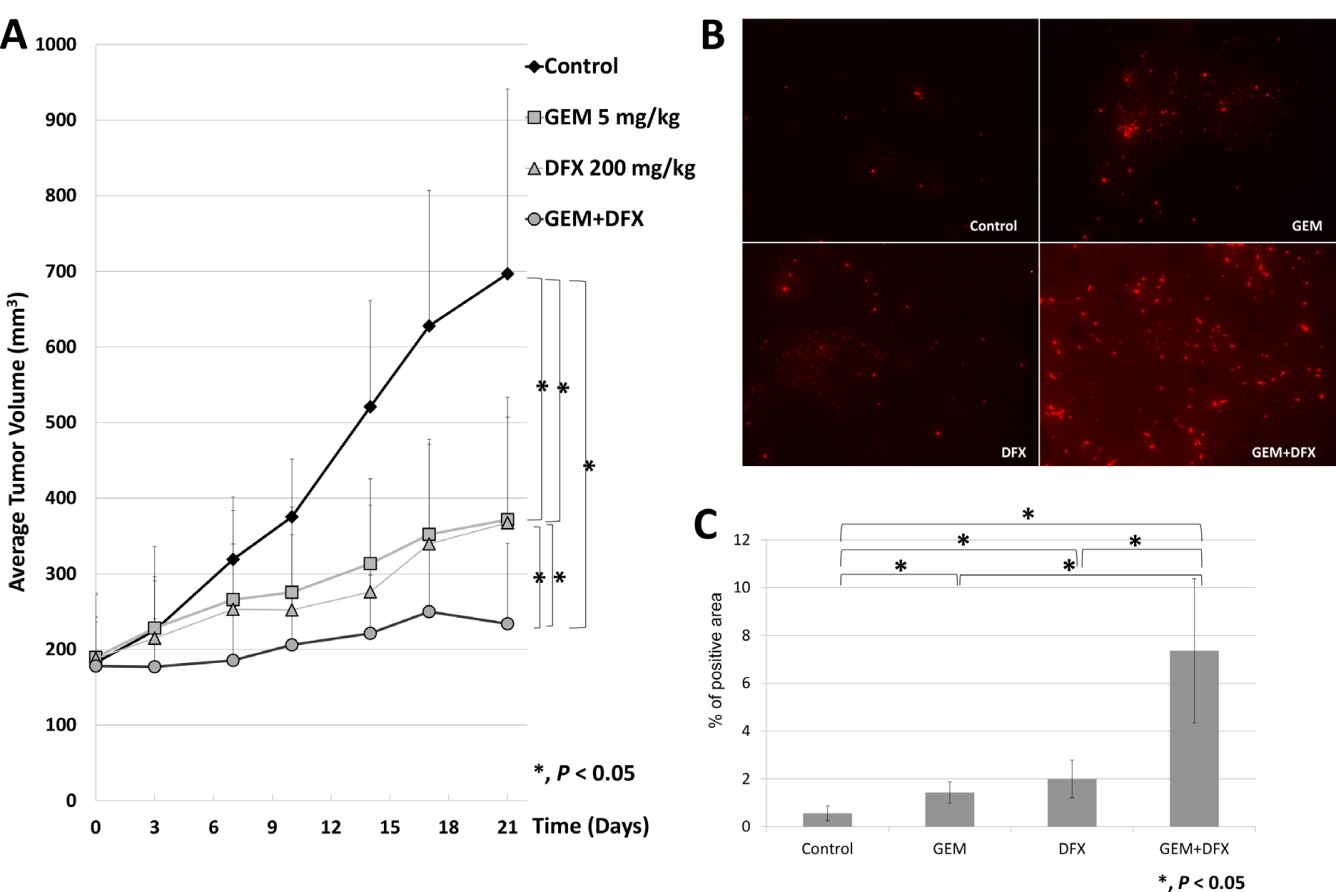
GEM+DFX suppressed tumor growth and induced apoptosis without any serious side effects *in vivo* (**A**) Data are presented as mean ± SD. The average tumor volumes of the mice treated with vehicle control, GEM, DFX, and GEM+DFX were 697 ± 244, 372 ± 136, 372 ± 166, and 234 ± 107 mm^3^, respectively. The mice treated with GEM+DFX had a significantly suppressed xenograft tumor growth. (Control, *n* = 10; GEM, *n* = 12; DFX, *n* = 10; and GEM+DFX, *n* = 12). (**B**) Tumor cells were assessed for apoptosis using a TUNEL kit, which labels apoptotic nuclei with a fluorescent maker. (Magnification, ×200). (**C**) Tumor cells from the mice treated with GEM+DFX showed an obvious increase in apoptosis. The TUNEL stain-positive area was counted using Dynamic cell count BZ-HIC software (model BZ-9000; Keyence Co., Osaka, Japan).

The antiproliferative activity of the combined treatment of GEM+DFX against pancreatic cancer cells was assessed *in vivo* using BxPC-3 pancreatic cancer xenografts in BALB/c nude mice. The average tumor volumes of the control, GEM, DFX, and GEM+DFX groups were 697 ± 244, 372 ± 136, 372 ± 166, and 234 ± 107 mm^3^, respectively (Figure 3A). A significantly suppressed xenograft tumor growth without serious side effects, such as weight loss or altered serum biochemistries (albumin, AST, ALT, Cre, and AMY), was observed in the GEM+DFX group (Table 1). Moreover, in the blood sample examinations, the DFX and GEM+DFX groups had significantly decreased serum ferritin levels (16.0 ± 4.1 and 14.6 ± 3.8 ng/ml, respectively) compared with the control (26.8 ± 15.1 ng/ml) and GEM (26.1 ± 9.4 ng/ml) groups (Table1). Tumor cells were assessed for apoptosis using a terminal deoxynucleotidyl transferase-deoxyuridine triphosphate nick-end labeling (TUNEL) kit, which labels apoptotic nuclei with a fluorescent maker. As shown in Figure 3B and 3C, tumor cells in the GEM+DFX group had a significantly increased apoptosis.

**Table 1:** Body weight of and serum biochemistries from nude mice

		Treatment groups			
	Units	Control	GEM	DFX	GEM+DFX
Body weight	g	21.8 ± 2.5	22.2 ± 2.0	22.4 ± 1.7	22.3 ± 2.3
Serum biochemistry					
Ferritin	ng/ml	26.8 ± 15.1	26.1 ± 9.4	16.0 ± 4.1^a,b^	14.6 ± 3.8^a,b^
Albumin	g/dl	3.6 ±0.2	3.6 ± 0.2	3.8 ± 0.1	3.8 ± 0.1^a,b^
Aspartate aminotransferase	U/l	141.7 ± 44.6	147.3 ± 36.1	132.8 ± 37.4	139.2 ± 28.5
Alanine transaminase	U/l	25.9 ± 9.4	26.3 ± 10.2	27.0 ± 7.1	30.9 ± 8.5
Creatinine	mg/dl	0.13 ± 0.05	0.13 ± 0.04	0.10 ± 0.00	0.10 ± 0.00^a^
Amylase	U/l	907.5 ± 146.0	841.7 ± 74.9	894.8 ± 139.8	951.9 ± 112.2

^a^*p* < 0.05 vs control.

^b^*p* < 0.05 vs GEM.

Mice treated with GEM+DFX had significantly suppressed xenograft tumor growth without serious side effects, such as weight loss or altered serum biochemistry. Mice treated with DFX and GEM+DFX had significantly decreased serum ferritin levels compared with those treated with the vehicle control and GEM.

### Suppression of RRM1 and RRM2 protein expression by DFX

**Figure 4:**
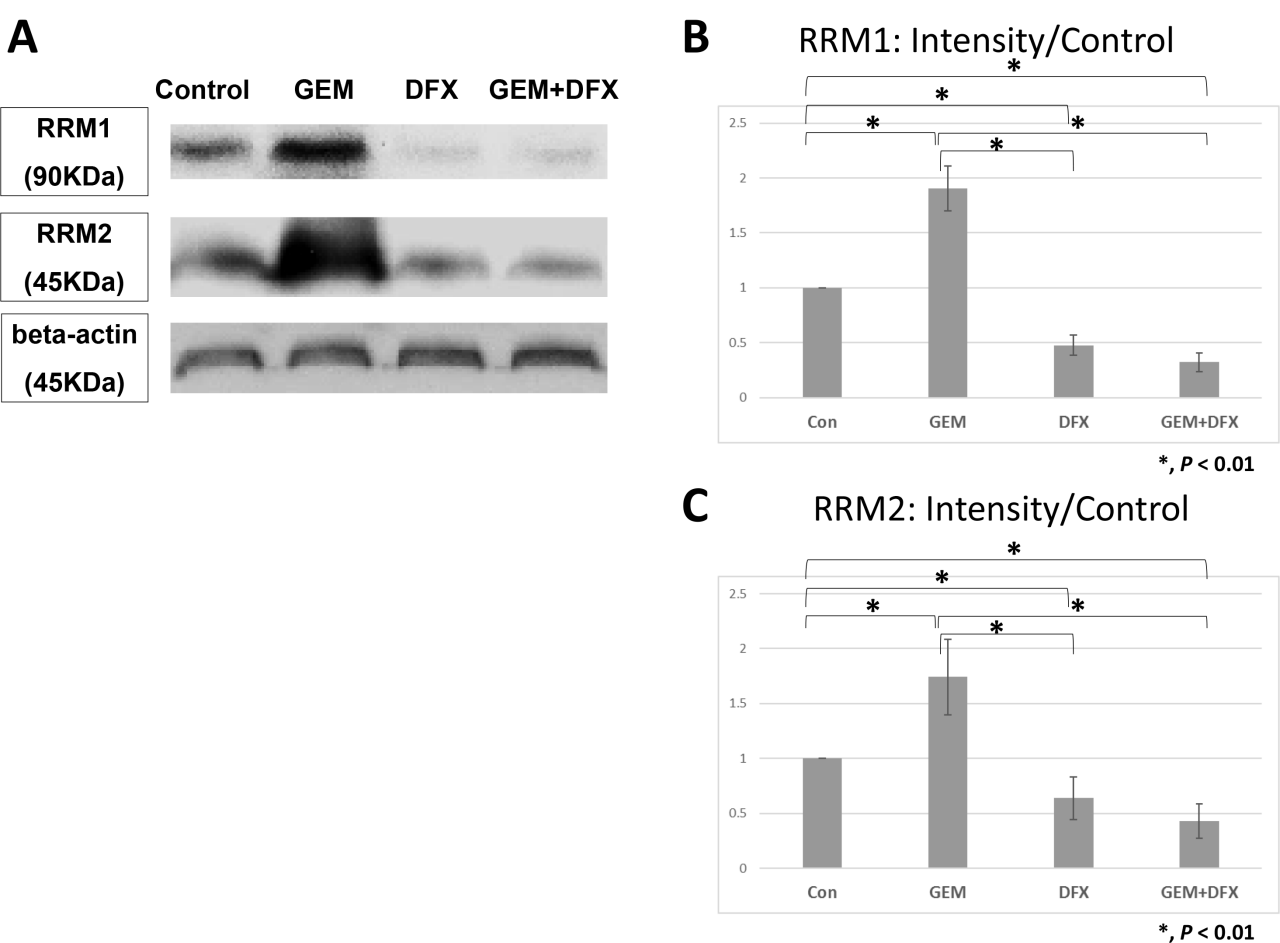
DFX suppressed RRM1 and RRM2 protein expression levels The average RRM1 protein band intensity (intensity/control) of the GEM, DFX, and GEM+DFX groups were 1.90 ± 0.20, 0.48 ± 0.09, and 0.32 ± 0.08, respectively. The average RRM2 protein band intensity (intensity/control) of the GEM, DFX, and GEM+DFX groups were 1.74 ± 0.34, 0.64 ± 0.19, and 0.43 ± 0.16, respectively. RRM1 and RRM2 expression levels significantly upregulated in the cells treated with GEM, but were significantly downregulated in the cells treated with DFX and GEM+DFX. Data are presented as mean ± SD (*n* = 4).

Ribonucleotide reductase (RR) subunit 1 (RRM1) and RR subunit 2 protein levels in BxPC-3 cells were assessed by western blot. The average RRM1 protein band intensity (intensity/control) of the GEM, DFX, and GEM+DFX groups were 1.90 ± 0.20, 0.48 ± 0.09, and 0.32 ± 0.08, respectively. The average RRM2 protein band intensity (intensity/control) of the GEM, DFX, and GEM+DFX groups were 1.74 ± 0.34, 0.64 ± 0.19, and 0.43 ± 0.16, respectively. The RRM1 and RRM2 protein expression levels were significantly upregulated in the cells treated with GEM, but were significantly down-regulated in the cells treated with DFX and GEM+DFX (Figure 4A–4C). These results showed that RRM1 and RRM2 protein levels were substantially reduced by DFX in BxPC-3.

### Panc-1 showed same trend as BxPC-3 in antiproliferative activity and RRM1 protein expression levels

**Figure 5:**
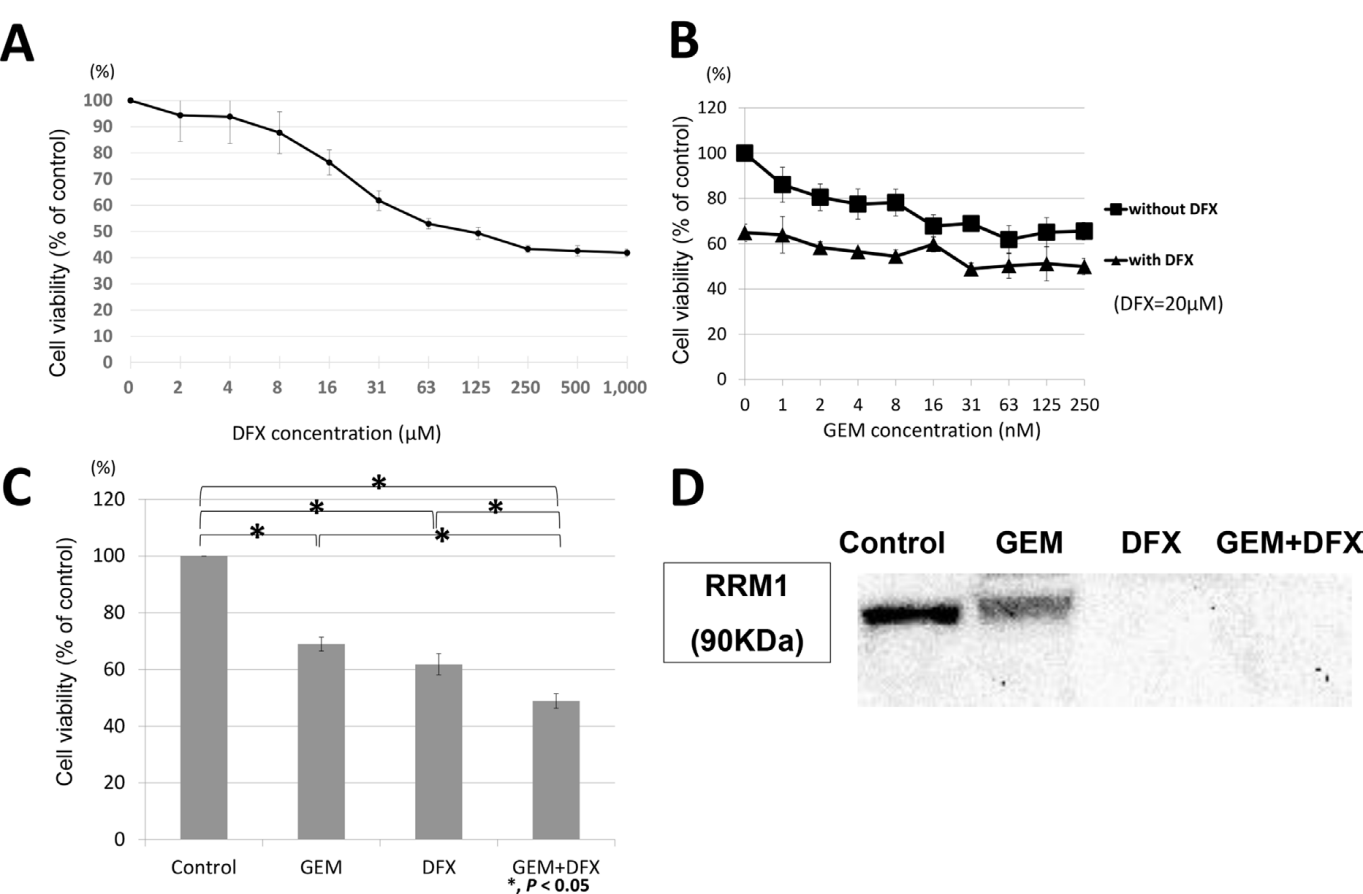
Panc-1 showed same trend as BxPC-3 in antiproliferative activity and RRM1 protein expression levels (**A**) DFX inhibited the proliferation of Panc-1 cells. The cells were treated with DFX for 72 h. The viability of Panc-1 cells incubated with DFX decreased in a dose-dependent manner. Data are presented as mean ± SD (*n* = 3). (**B**) GEM and GEM+DFX inhibited the proliferation of Panc-1 cells. The cells were treated with GEM and/or DFX for 72 h. The viability of Panc-1 cells incubated with GEM and/or DFX decreased in a dose-dependent manner. Data are presented as mean ± SD (*n* = 3). (**C**) The antiproliferative activity of the combined treatment of 20 nM GEM and 20 μM DFX for Panc-1 was significantly higher than that of GEM alone. Data are presented as mean ± SD (*n* = 3). (**D**) RRM1 protein expression levels of Panc-1 were down-regulated in the cells treated with DFX and GEM+DFX. But the RRM1 protein expression levels were also slightly down-regulated in the cells treated with GEM.

Panc-1, another cell line, was also evaluated. Panc-1 was also incubated with either the vehicle control or the indicated concentrations of DFX for 72 h; subsequently, cell survival rates were measured using the MTS assay. As shown in Figure 5A, the IC50 value of DFX in Panc1 was 23.4 ± 3.7 μM. Thus, Panc-1 was exposed to GEM (0, 1, 2, 4, 8, 16, 31, 63, 125, 250 nM) with 20 μM DFX or without DFX for 72 h. The cell survival rates are shown in Figure 5B. The antiproliferative activity of the combined treatment of 20 nM GEM and 20 μM DFX was significantly higher than that of GEM alone (Figure 5C). RRM1 protein levels in Panc-1 cells were also assessed by western blot. RRM1 protein expression levels of Panc-1 were downregulated in the cells treated with DFX and GEM+DFX (Figure 5D). These results showed that Panc-1 also followed same trend as BxPC-3.

## DISCUSSION

GEM still remains a key drug for pancreatic cancer today; however, the most important problem is gemcitabine resistance. Many studies have tackled gemcitabine resistance [10]. The levels of GEM’s active derivate gemcitabine triphosphate must comprise a sufficient proportion of the cellular pool of deoxynucleotides (dNTPs) to be efficiently incorporated into the DNA. Expansion of naturally occurring dNTPs in the nucleotide pool leads to GEM resistance. Moreover, RR plays an essential role in the maintenance of the deoxyribonucleotide pool, and RR upregulation also leads to GEM resistance [11, 12]. This resistance mechanism was shown to be clinically relevant in lung [13, 14] and breast [15] cancer. In pancreatic cancer, interestingly, RRM1 levels were inversely correlated with patient survival [16].

The antiproliferative activity of iron chelators was first demonstrated in leukemia in 1986 [17, 18], and the antiproliferative activity of DFX has been investigated in various cancers [7, 8, 19–22].

Iron chelators are known to have antiproliferative effects in cancer by RR inactivation [23]. RR catalyzes the rate-limiting step in DNA synthesis, which is the reductive conversion of ribonucleotides to deoxyribonucleotides [5]. RR is constructed from large RRM1 and small RRM2 subunits. RRM1 is constantly expressed throughout the cell cycle [24], while RRM2 is initiated during S-phase [24] and is degraded following M-phase [25]. The catalytic activity of RR is dependent on a dinuclear iron site in the RRM2 subunit; RRM2 requires iron for stabilization [26]. Thus, RR regulation involves the control of RRM1 and RRM2 activity and expression [27]. The absence of a constant supply of iron to RRM2 results in RRM1 inactivation [2]. Moreover, a previous study reported a synergistic effect between hydroxyurea, a RR inhibitor, and gemcitabine on gemcitabine-resistant cells [28]. However, the effect of iron chelators on RRM1 and RRM2 expression has not been studied in detail [27].

To address gemcitabine resistance and pancreatic cancer cell proliferation, we conducted an *in vitro* and *in vivo* study to assess the antiproliferative activity of the combined treatment of GEM+DFX. We found that GEM+DFX could inhibit pancreatic cancer cell proliferation and induce apoptosis *in vitro* and *in vivo*. Moreover, we confirmed that RRM1 and RRM2 protein expressions in cells incubated with DFX and GEM+DFX were significantly lower than those in the control cells *in vitro*.

**Figure 6:**
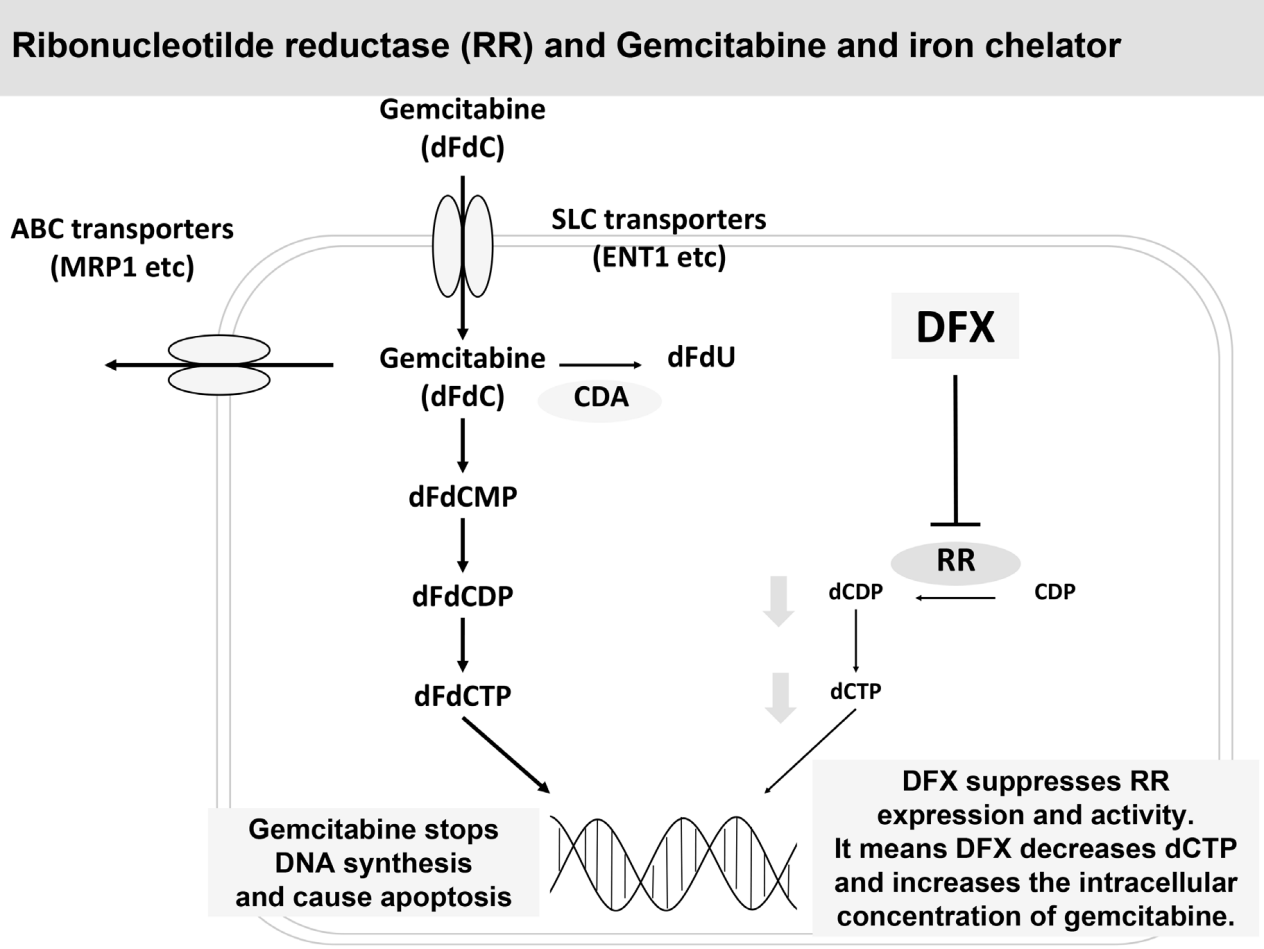
DFX suppressed RR expression and activity and potentiated the effect of GEM DFX not only demonstrated antiproliferative activity by suppressing RR expression and activity but also potentiated the effect of GEM by decreasing the competition between GEM and deoxycytidine.

Therefore, DFX not only demonstrated antiproliferative activity by suppressing RR expression and activity but also potentiated the effect of GEM by decreasing the competition between GEM and deoxycytidine triphosphate (Figure 6). Moreover, as iron chelators are not classified as anticancer drugs and are primarily used for iron-overload disease, GEM+DFX showed therapeutic advantages without serious side effects over single-agent GEM. Considering that more than half of patients with pancreatic cancer are diagnosed at an age of ≥65 years [1], treatment with GEM+DFX possibly has favorable outcomes in clinical chemotherapy. Furthermore, this therapeutic method might turn out to be applied to other cancer like non-small cell lung cancer, ovarian cancer and bladder cancer in future. In addition, combination chemotherapies, such as regimens combining DFX, GEM, and albumin-bound paclitaxel, possibly have therapeutic advantages over albumin-bound paclitaxel with GEM in pancreatic cancer. Furthermore, a synergistic effect between DFX and fluorouracil, used in FOLFIRINOX on pancreatic cancer, in pancreatic cancer could be expected.

In conclusion, the combination therapy using GEM+DFX has significant anticancer effects through the suppression of RR activity by DFX without any serious side effects, which in turn indicates the potential of this new pancreatic cancer therapy using iron chelators and GEM as well as iron chelators and antimetabolites.

## MATERIALS AND METHODS

### Cell culture

We reported that DFX shows antiproliferative activity against BxPC-3, a pancreatic cancer cell line [8]. In this study, we also used BxPC-3 and Panc-1 obtained from the American Type Culture Collection (Manassas, VA, USA). BxPC-3 and Panc-1 are an epithelial cell line derived from pancreatic adenocarcinoma. BxPC-3 cells were grown in RPMI-1640 (Life Technologies, Carlsbad, CA, USA) with 10% (v/v) fetal calf serum and 50 μg/ml gentamicin. Panc-1 cells were grown in Dulbecco’s Modified Eagle’s Medium (Life Technologies) with 10% (v/v) fetal calf serum and 50 μg/ml gentamicin. All cells were incubated at 37° C in a humidified atmosphere containing 5% CO_2_ [8].

### Reagents

GEM (Gemzar) was purchased from Eli Lilly and Co. (Indianapolis, IN, USA), while the oral iron chelator DFX was purchased from Novartis (Basel, Switzerland). For *in vitro* studies, GEM and DFX were used by dilution in culture media containing 10% fetal calf serum. For *in vivo* studies, GEM was dissolved in 15% propylene glycol/0.9% saline, while DFX was dissolved in a sodium chloride solution (0.9% w/v; Chemix Inc., Shinyokohama Kohoku-ku, Yokohama, Japan) [8].

### Cell proliferation

Cellular proliferation was evaluated using the MTS assay. Cell suspensions (3000 cells/100 μl) were added to each well in a 96-multiwell culture plate (BD Bioscience, San Jose, CA, USA) and incubated at 37° C for 24 h. GEM (0, 5, 10, 20, 39, 78, 156, 312 nM) and/or DFX (0, 1, 2, 4, 8, 16, 31, 63, 125, 250, 500, 1000 μM) was subsequently added to each well, and the cells were incubated for a further 72 h. At the end of the culture period, 10 μl of MTS solution (Promega, Madison, WI, USA) was added to each 100 μl of culture media, which were incubated for 2 h. Absorbance at 490 nm was measured with a multimode reader (Infinite 200 PRO, Tecan Trading, AG, Switzerland), and the results were expressed as the percentage viable with respect to the untreated control [8, 19].

### Apoptosis analysis by luminescence assay and flow cytometry

For apoptosis analysis, cell suspensions (3000 cells/100 μl) were added to each well in a 96-multiwell culture plate (BD Bioscience) and incubated at 37° C for 24 h. GEM (20 nM) and/or DFX (20 μM) was added to each well, and the cells were incubated for a further 72 h. After harvesting, caspase activity was measured using the caspase 3/7 assay kit (Caspase-Glo 3/7 kit, Promega) according to the manufacturer’s instructions. Apoptosis was evaluated with an apoptosis detection kit (Annexin V Apoptosis Detection Kit APC, eBioscience, San Diego, CA, USA) according to the manufacturer’s instructions. After staining, the cells were examined using a flow cytometer (Gallios, Beckman Coulter, Fullerton, CA, USA). The data were analyzed by FlowJo software (Tree Star, Inc., Ashland, OR, USA) [8, 19].

### Tumor xenografts in nude mice

Animal care was performed in accordance with the animal ethics requirements at Yamaguchi University School of Medicine, and the experimental protocol was approved by the institutional animal care and user uommittee (approval ID 21-035). Female BALB/c (nu/nu) mice were purchased from Nippon SLC (Shizuoka, Japan) and were housed in sterile conditions. Experiments commenced when the mice were 8-10 weeks of age. BxPC-3 cells in culture were harvested and resuspended in a 1:1 ratio of RPMI-1640 and Matrigel (BD Bioscience). Viable cells (3 × 10^6^ cells) were administered subcutaneously into the back of the mice.

After engraftment, tumor size was measured using vernier calipers every 2 days, and tumor volume (TV; mm^3^) was calculated using the following formula: TV = d^2^ × D/2, where d and D are the shortest and the longest diameter, respectively. When the average TV reached approximately 180 mm^3^, drug administration began (day 0).

The mice were divided into four groups: control (*n* = 10), GEM (*n* = 12), DFX (*n* = 10), and GEM+DFX (*n* = 12) groups. The treatment administration schedule was based on our past study [8] and a previous study [29]. The DFX and GEM+DFX groups received DFX, which was administered by oral gavage every 2 days, with three treatments per week, for 21 days at a concentration of 200 mg/kg. The control and GEM groups received vehicle alone (PBS), which was also administered by oral gavage. The GEM and GEM+DFX groups received GEM, which was administered intraperitoneally every 3 days, with two treatments per week, for 21 days at a concentration of 5 mg/kg. The control and DFX groups received vehicle alone (PBS), which was also administered intraperitoneally.

At the end of the experiment, the mice were sacrificed, and the tumors were excised and processed for TUNEL staining. Blood samples were collected simultaneously during tumor removal. Serum ferritin levels were measured using the enzyme-linked immunoassay method (Mouse Ferritin ELISA kit, Kamiya Biochemical Company, Seattle, WA, USA). Serum biochemistries other than ferritin were analyzed by Yamaguchi Laboratory Co., Ltd. (Ube, Japan) [8].

### Histology and TUNEL staining

Tumor sections (3-mm-thick) were fixed in 4% paraformaldehyde (Muto Pure Chemicals, Tokyo, Japan) for 72 h and paraffin embedded. Apoptotic tumor cells were detected by TUNEL staining using an *In Situ* Detection kit (TMR Red; Roche Diagnostics, Indianapolis, IN, USA) according to the manufacturer’s protocol. The TUNEL stain-positive area was measured using Dynamic cell count BZ-HIC software (model BZ-9000; Keyence Co., Osaka, Japan).

### Western blot analysis

BxPC-3 and Panc-1 cells were treated with 20 nM of GEM and/or 20 μM of DFX for 72 h. The cells were homogenized in lysis buffer on ice. Suspensions were incubated for 1 h at 4° C and centrifuged at 15,000 × g for 30 min at 4° C. The collected supernatants were used for western blotting after protein concentration measurement using the Lowry method [30]. The samples from BxPC-3 cells were prepared three times independently.

For western blotting, 40 μg of protein was used. Sodium dodecyl sulfate-polyacrylamide gel electrophoresis was performed in pre-cast gels (12% acrylamide; Mini-PROTEAN TGX Gels, Bio-Rad, Hercules, CA, USA). The primary antibodies were as follows: rabbit monoclonal antibody against ribonucleotide reductase (RR) subunit 1 (RRM1, dilution 1:3000, ab137114; Abcam, Cambridge, MA, USA), rabbit monoclonal antibody against RR subunit 2 (RRM2, dilution 1:1500, ab172476; Abcam), and mouse monoclonal antibody against beta-actin (dilution 1:10000, ab6276; Abcam). Membranes were incubated with the primary antibodies overnight at 4° C, washed three times with PBS containing 0.05% Tween-20 and once with PBS, and incubated with a horseradish peroxidase-conjugated secondary antibody (dilution 1:5000; GE Healthcare, Buckinghamshire, England, UK) for 1 h at room temperature [31]. Protein bands were quantitated by densitometric analysis using image analysis software (Quantity One; BioRad, Hercules, CA, USA).

### Statistical analysis

Statistical significance was determined using Student’s *t* test or analysis of variance. JMP 13 statistical software (SAS Institute Inc., Cary, NC, USA) was employed in the analysis. Results are expressed as mean ± standard deviation (SD), and differences with *p* < 0.05 were considered statistically significant.
